# CSK-mediated signalling by integrins in cancer

**DOI:** 10.3389/fcell.2023.1214787

**Published:** 2023-07-07

**Authors:** Horacio Maldonado, Lisette Leyton

**Affiliations:** ^1^ Receptor Dynamics in Cancer Laboratory, Institute of Systems, Molecular and Integrative Biology, University of Liverpool, Liverpool, United Kingdom; ^2^ Cellular Communication Laboratory, Programa de Biología Celular y Molecular, Center for Studies on Exercise, Metabolism and Cancer (CEMC), Instituto de Ciencias Biomédicas (ICBM), Facultad de Medicina, Universidad de Chile, Santiago, Chile; ^3^ Advanced Center for Chronic Diseases (ACCDiS), Faculty of Chemical and Pharmaceutical Sciences and Faculty of Medicine, Universidad de Chile, Santiago, Chile

**Keywords:** cancer cells, signal transduction, integrins, Src family kinases, cell contraction, mechanobiology

## Abstract

Cancer progression and metastasis are processes heavily controlled by the integrin receptor family. Integrins are cell adhesion molecules that constitute the central components of mechanosensing complexes called focal adhesions, which connect the extracellular environment with the cell interior. Focal adhesions act as key players in cancer progression by regulating biological processes, such as cell migration, invasion, proliferation, and survival. Src family kinases (SFKs) can interplay with integrins and their downstream effectors. SFKs also integrate extracellular cues sensed by integrins and growth factor receptors (GFR), transducing them to coordinate metastasis and cell survival in cancer. The non-receptor tyrosine kinase CSK is a well-known SFK member that suppresses SFK activity by phosphorylating its specific negative regulatory loop (C-terminal Y^527^ residue). Consequently, CSK may play a pivotal role in tumour progression and suppression by inhibiting SFK oncogenic effects in several cancer types. Remarkably, CSK can localise near focal adhesions when SFKs are activated and even interact with focal adhesion components, such as phosphorylated FAK and Paxillin, among others, suggesting that CSK may regulate focal adhesion dynamics and structure. Even though SFK oncogenic signalling has been extensively described before, the specific role of CSK and its crosstalk with integrins in cancer progression, for example, in mechanosensing, remain veiled. Here, we review how CSK, by regulating SFKs, can regulate integrin signalling, and focus on recent discoveries of mechanotransduction. We additionally examine the cross talk of integrins and GFR as well as the membrane availability of these receptors in cancer. We also explore new pharmaceutical approaches to these signalling pathways and analyse them as future therapeutic targets.

## 1 Introduction

Cancer is a complex and multifactorial disease that remains a significant public health issue worldwide. The abnormal growth and proliferation of cells that can invade and spread to other parts of the body are distinctive features of cancer progression, leading to the destruction of healthy tissues and organs (Hanahan and Weinberg, 2000). The molecular mechanisms underlying cancer progression and metastasis are complex and heavily controlled by various signalling pathways, including the integrin family receptors (Hanahan and Weinberg, 2000; Cooper and Giancotti, 2019).

**FIGURE 1:**
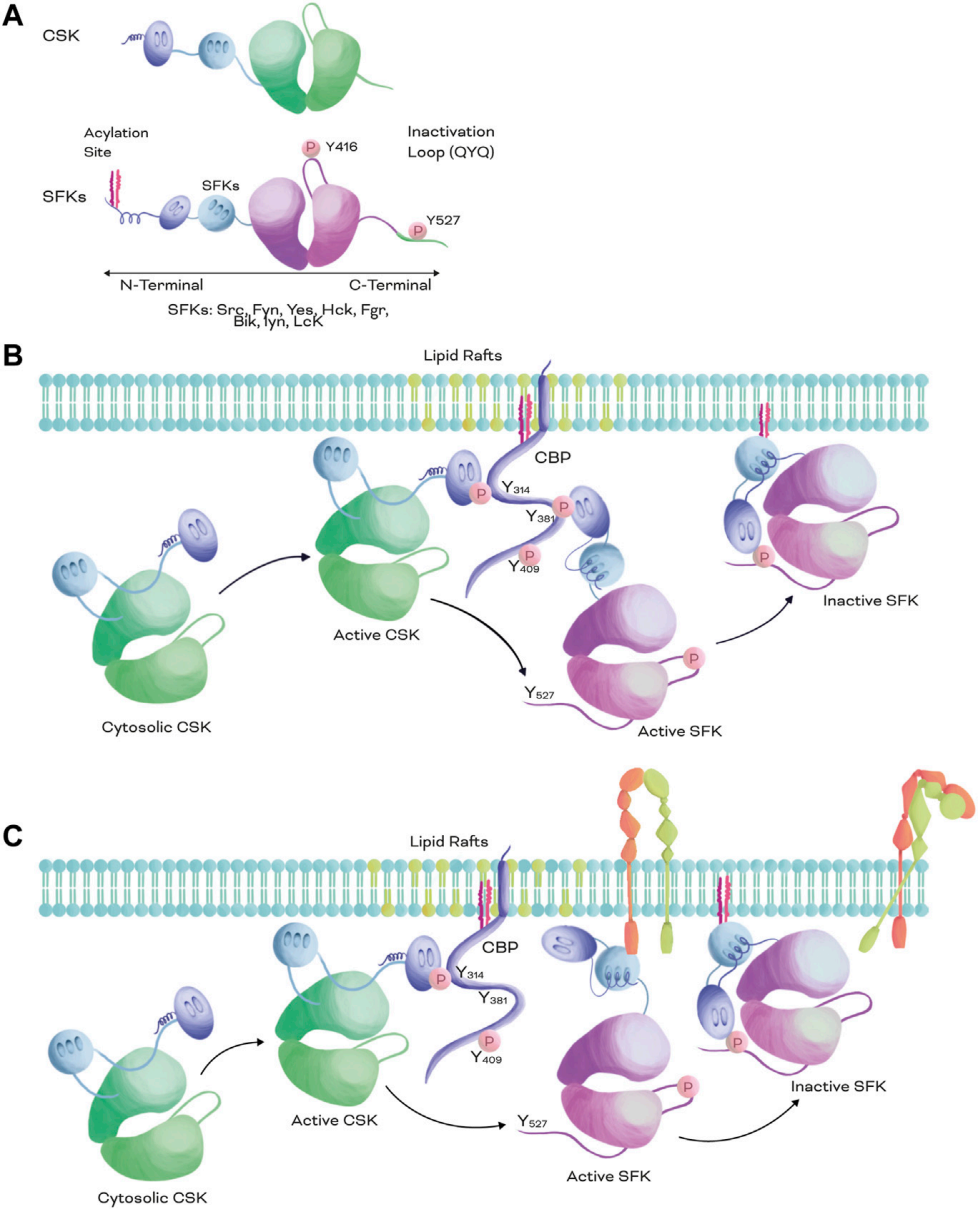
Structural comparison of SFKs and CSK and inactivation mechanism of SFKs. **(A)**. Structural representation of CSK (top) and SFKs (bottom). Both CSK and SFKs possess a kinase catalytic domain (green and pink, respectively), a SH3 domain (light blue), and a SH2 domain (purple). However, SFKs also possess an acylation site at the N-terminal and a negative regulatory phosphorylation at the C-terminal. **(B,C)**. SFKs inactivation mechanism. When CSK is recruited to the plasma membrane from the cytosol by scaffolding or adapter proteins, such as CBP, CSK is fully activated and phosphorylates the negative regulatory loop of SKFs at Y^527^ or equivalent QYQ peptide sequence, promoting a closed conformation and excluding the inactive kinase from the lipid rafts. When SFKs are activated, they can be recruited to the same scaffolding protein than CSK **(B)** or bound to other membrane receptors such as integrins **(C)**. The membrane proximity of CSK and SFKs promotes the Y^527^ phosphorylation of SFKs **(B,C)** enabling an efficient inactivation of the kinase and the receptor **(C)**.

Integrins are cell adhesion molecules that play a vital role in regulating key biological processes, such as cell migration, invasion, proliferation, and survival. Focal adhesions, which are mechanosensing complexes that connect the extracellular environment with the cell interior through integrin receptors, are key players in cancer progression (Cooper and Giancotti, 2019; Kechagia et al., 2019; Harburger and Calderwood, 2009). The Src family kinases (SFKs) are known to interact with integrins and downstream effectors and can integrate extracellular cues sensed by integrins and growth factor receptors (GFRs) (Thomas and Brugge, 1997; Harburger and Calderwood, 2009). These interactions coordinate metastasis and cell survival in cancer (Hamidi et al., 2016; Hamidi and Ivaska, 2018). The SFKs include the non-receptor tyrosine kinase C-terminal Src kinase (CSK), which plays a pivotal role in tumour progression and suppression by inhibiting SFK oncogenic effects in several cancer types (Okada et al., 1991; Nakagawa et al., 2000; Cui et al., 2019). Despite extensive research on SFK oncogenic signalling, the specific role of CSK and its crosstalk with integrins in cancer progression, for example, in mechanosensing, remains unclear. Src and other members of the SFK possess a lipid moiety that allow them to localize at the inner membrane of the lipid bilayer (Okada, 2012). When integrins get activated, they can interact directly with SFK or indirectly via other proteins in the focal adhesions. However, CSK is an exception of the SFK because it lacks this lipid anchor moiety; therefore, to localize at the plasma membrane proximity, CSK needs to interact with adaptor or scaffold proteins, such as Paxillin or the CSK-binding protein (CBP) (Figure 1) (Kawabuchi et al., 2000; Okada, 2012).

This article aims to review recent advances in our understanding of integrin signalling and the role of SFKs and CSK in cancer progression. In the next sections we will discuss integrin function, the crosstalk between integrins and SFKs relevant for cancer progression, and finally, how CSK can regulate integrin functions in cancer through the modulation of SFK signalling.

## 2 Integrin structure and function

Integrins are sensors of the cellular microenvironment, connecting the extracellular matrix (ECM) with the cell interior (Harburger and Calderwood, 2009; Chastney et al., 2021). They control central and diverse cellular functions, including migration, survival, differentiation, adhesion, and ECM deposition (Schwartz, 2010; Chastney et al., 2021). Integrins can bind both ECM (such as Fibronectin, collagens, laminins, etc.), or other cell adhesion molecules (e.g., Thy-1, syndecans, L1CAM, VCAM1, and ICAM1) present on the membrane surface of the same cell (cis) or other cells (trans), triggering cell signalling in both (Humphries et al., 2006; Hermosilla et al., 2008).

Integrins are heterodimeric transmembrane receptors with an alpha and a beta subunit (Chastney et al., 2021). In mammals, there are 24 possible combinations of alpha and beta subunits. We can classify these integrin dimers in 4 sub-groups according to the specific component of ECM/protein that they bind in the extracellular environment: 1. Collagen-binding receptors (α1β1, α2β1, α10β1, and α11β1) (Heino, 2014; Zeltz and Gullberg, 2016), 2. RGD-binding receptors that can bind RGD (or RDG-like) peptide sequences present in Vitronectin, Fibronectin, Fibrinogen, Thy-1, and the Latency-associated peptide (αVβ1, αVβ3, αVβ5, αVβ6, αVβ8, α5β1, α8β1, and αIIbβ3) (Thomas et al., 2002; Maldonado et al., 2017; Benito-Jardón et al., 2020; Shen et al., 2021), 3. Laminin binding receptors (α3β1, α6β1, α7β1, and α7β4) (Arimori et al., 2021), and 4. Leukocyte specific receptors (α9β1, α4β1, α4β7, αEβ7, αLβ2, αMβ2, αXβ2, and αDβ2) (Evans et al., 2009; Hyun et al., 2009; Mambole et al., 2010; Lamb et al., 2016; Jawhara et al., 2017; Cluny et al., 2022).

Integrins do not possess intrinsic enzymatic activity, but can directly or indirectly link via their intracellular domain with adapter proteins and proteins that possess enzymatic activity, such as kinases, phosphatases and small GTPases (via adapter proteins) (Kechagia et al., 2019; Chastney et al., 2021). When integrins bind to their ligands, they initiate signalling from the extracellular to the intracellular environment, which is known as outside-in signalling. This enables cells to perceive and respond to signals from its surroundings. For example, Integrin engagement regulates activation of the Rho family of small GTPases, which controls actin filament dynamics via proteins such as ROCK, PAK, Myosin, and Arp2/3, among others (Schwartz and Shattil, 2000; Huveneers and Danen, 2009a). Integrins can also sense and respond to signals from within the cell, influencing events in the extracellular environment through a process called inside-out signalling (Harburger and Calderwood, 2009). For instance, the interaction between Talin and Kindlin with the cytoplasmic domain of the integrin β-chain facilitates the binding of integrins to extracellular ligands (Humphries et al., 2006; Harburger and Calderwood, 2009; Schwartz, 2010; Horton et al., 2016).

Integrins are crucial components of focal adhesion complexes; as such, they can regulate focal adhesion dynamics by recruiting/excluding proteins from the adhesion sites, such as focal adhesion kinase (FAK), SFKs, Paxillin, Talin, and Vinculin, among others (Horton et al., 2016; Legerstee et al., 2019). The protein machinery recruited to Integrin adhesion complexes (IACs) that connect integrins and the actin cytoskeleton is known as the “Integrin adhesome” (Winograd-Katz et al., 2014; Horton et al., 2015; Horton et al., 2016). The term “adhesome” was introduced to emphasize the intricate interconnectedness and complexity of the proteins involved in cell adhesion. It provides a comprehensive view of cell adhesion, highlighting the integrated nature of these molecular components and their coordinated actions in regulating cellular behaviour.

Within the adhesome, integrin-binding and actin-binding protein complexes mechanically couple integrins to the actomyosin system and effectively act as a “transmission system,” which facilitates the transfer of forces and can be regulated similarly to a “molecular clutch” (Schwartz, 2010; Cooper and Giancotti, 2019; Wang et al., 2019; Chastney et al., 2021). This dynamic regulation allows integrins to finely control processes, such as cell adhesion, migration, and ECM composition (Schwartz and DeSimone, 2008; Charras and Sahai, 2014).

Integrins can transmit forces from the ECM to the cell interior, allowing cells to sense ECM stiffness and modifying the architecture of the surrounding microenvironment (Cooper and Giancotti, 2019; Kechagia et al., 2019). ECM stiffness/rigidity can be defined as the inherent physical property of the ECM to resist deformation when exposed to applied forces (Stiffness, 2000; Park et al., 2023). Several factors contribute to ECM stiffness, including composition (expression and amount of ECM proteins), architecture (how those proteins are arranged), and elasticity (Frantz et al., 2010; Chaudhuri et al., 2014). Proteins such as collagen, elastin, Fibronectin, and Laminin contribute to the mechanical strength and elasticity of the ECM. Cross-linking of these proteins also plays a role in determining overall stiffness (Frantz et al., 2010). The engagement of integrins with the ECM components allows sensing and regulating the architecture and stiffness of the ECM by activating pathways that regulate ECM deposition or degradation (Kechagia et al., 2019; Barber-Pérez et al., 2020). For example, α5β1 and αVβ3 integrins mediate expression and activation of metalloproteinases (MMPs), which degrade collagen fibres (Morozevich et al., 2009; Jiao et al., 2012).

ECM stiffness has significant implications for cell behaviour and tissue function. Cells can sense and respond to mechanical cues from their surrounding ECM in processes known as mechanosensing and mechanotransduction, respectively (Schwartz and DeSimone, 2008; Chen et al., 2017; Martino et al., 2018). Changes in ECM stiffness can affect cell adhesion, migration, proliferation, differentiation, metabolism, and gene expression (Zaman et al., 2006; Kim et al., 2018; Barber-Pérez et al., 2020; Ge et al., 2021; Li et al., 2021). Stiffness of the ECM can vary between different tissues and during both physiological and pathological conditions (Handorf et al., 2015; Timraz et al., 2015; Yi et al., 2022). For instance, stiffening of the ECM is often associated with fibrosis, tumor progression, and aging (Jiang et al., 2022; Guo et al., 2022; Segel et al., 2019; Fournet et al., 2018; Elgundi et al., 2019; Seewaldt, 2014; Wullkopf et al., 2018).

Integrins can interact and regulate activation of growth factors in the extracellular compartment. AlphaV dimers, including αVβ1, αVβ3, αVβ5, αVβ6, and αVβ8, interact with latent TGFβ via the RGD-binding sequence (Munger et al., 1999; Shi et al., 2011; Malenica et al., 2021). TGFβ is one of the main drivers of cancer progression and is secreted in latent complexes (Derynck et al., 2021). Upon binding the RGD peptide (Latency Associated Peptide, LAP) sequence in the latent complex, integrins apply force via cellular contraction, deforming the complex and releasing active TGFβ from the latent complex into the extracellular compartment (Shi et al., 2011). Thus, integrin signalling dysregulation directly impacts cell motility, ECM composition, and growth factor activation via TGFβ regulation (Colak and Ten Dijke, 2017; Brown and Marshall, 2019).

In cancer cells, neoplastic conversion alters the expression of specific integrin heterodimers, impacting their downstream signalling and normal cellular functions (Hamidi and Ivaska, 2018). For example, αVβ6 and αVβ8 integrins are poorly expressed in healthy tissues; however, upon cell reprogramming in epithelial cells, they increase their expression in diverse cancer types, including breast cancer, lung adenocarcinoma, and colon cancer, among others (Yang et al., 2008; Reyes et al., 2013; Moore et al., 2014; Yan et al., 2018; Zhou et al., 2020). Most αV dimers can activate TGFβ *in vitro* (Munger et al., 1999; Annes et al., 2003). Nevertheless, the binding affinity constant between αVβ6/αVβ8 integrins and the LAP is an order of magnitude higher than for other integrin dimers, such as αVβ1, αVβ3, and αVβ5 (Buscemi et al., 2011; Shi et al., 2011; Ozawa et al., 2016; Rowedder et al., 2017). In addition, mice lacking αVβ6 and αVβ8, phenocopy TGF-β1 and TGF-β3 knockouts showing the relevance of those integrins in TGF-β1 activation (Aluwihare et al., 2009). Therefore, modifying the integrin repertoire may impact the levels of active TGFβ in the extracellular environment. Consequently, pro-oncogenic responses modulated by TGFβ, such as cell survival, invasion, apoptosis, cell adhesion, and motility, could also be dysregulated (Hamidi and Ivaska, 2018).

Other relevant downstream partners of integrins are SFKs (Huveneers and Danen, 2009b; Horton et al., 2016). When integrins bind to their ligands, they undergo conformational changes that result in integrin clustering and recruitment of SFKs to the cytoplasmic domain of integrins (outside-in signalling) (Shattil, 2005). This leads to the autophosphorylation and activation of SFKs at focal adhesions, enabling them to phosphorylate downstream targets and initiate signalling cascades (Shattil, 2005; Huveneers and Danen, 2009a). Additionally, SFKs can also regulate integrin activity through inside-out signalling. SFKs phosphorylate and activate FAK, which in turn phosphorylates several signalling molecules, including integrin cytoplasmic domain-associated protein-1 (ICAP-1) (Webb et al., 2004; Mitra and Schlaepfer, 2006). Phosphorylated ICAP-1 binds to the cytoplasmic domain of integrins, resulting in integrin activation and enhanced adhesion (Chang et al., 1997; Degani et al., 2002; Bouvard et al., 2006). SFKs can interact with other signalling molecules involved in integrin regulation, including small GTPases and tyrosine kinases (Clark et al., 1998; Schwartz and Shattil, 2000). For example, SFKs can phosphorylate and activate the non-receptor tyrosine kinase Pyk2 (proline-rich tyrosine kinase 2), which is implicated in integrin-mediated signalling (Sieg et al., 1998; Sanjay et al., 2001; Zhao et al., 2016). In summary, integrins and SFKs regulate each other bidirectionally. Integrins can activate SFKs through outside-in signalling, while SFKs can regulate integrin activity through inside-out signalling. This crosstalk between integrins and SFKs is crucial for coordinating cellular processes such as cell adhesion, migration, and signalling. In the next sections we will delve into the more detailed functions of SFKs and their interplay with integrins that are relevant for cancer progression.

## 3 Src family kinases

SFKs play an important role in regulating several cellular processes, including cell growth, differentiation, migration, and survival, among others. In mammals, this family of non-receptor tyrosine kinases includes 9 members: Src, Fyn, Lyn, Lck, Hck, Yes, Blk, Fgr, and its negative regulator CSK. SFKs, except for CSK, possess a very similar structural arrangement that includes SH2 and SH3 domains, a kinase domain, N-terminal fatty acylation sites and two regulatory phosphorylation sites at the C-terminus (Figure 1A) (Thomas and Brugge, 1997; Tatosyan and Mizenina, 2000). The phosphorylation of the first one at Y^416^ (for Src or equivalent, Figure 1A) usually occurs by autophosphorylation and increases the basal activity of the kinase domain; upon which SFKs turn into an open conformation. The second one at Y^527^ (for Src or an equivalent QYQ peptide sequence at C-terminus, Figure 1A) is phosphorylated by CSK and promotes intramolecular interaction between the SH2 domain and the C-terminus of SFKs, resulting in a closed conformation (inactive form) that does not allow the kinase domain to interact with substrates and relocates the SFKs (Figure 1B) (Howell and Cooper, 1994; Dias et al., 2022).

Proteins upstream of SFKs in the signalling cascades can be plasma membrane receptors or scaffolding proteins (Tatosyan and Mizenina, 2000). SFKs can directly or indirectly interact via their SH2 and SH3 domains with integrin intracellular domains (e.g., β1, β3, and β5, among others), GFRs (e.g., EGFR, HER2, PDGFR, FGFR, and others) (Figure 1C), and G protein coupled receptors (GPCRs) (Tatosyan and Mizenina, 2000; Luttrell and Luttrell, 2004; Shattil, 2005). They can also interact with scaffolding proteins located in lipid rafts (e.g., Paxillin and CBP) (Kawabuchi et al., 2000; Luttrell and Luttrell, 2004; Webb et al., 2004; Liu and Cowburn, 2016), controlling several cellular processes, such as apoptosis, proliferation, cell survival, cell differentiation, migration, cell adhesion, and protein trafficking. Therefore, the crosstalk mechanisms involving SFK activation, inhibition and localisation must be precisely regulated.

SFKs are known as proto-oncogenes, meaning that over-activation of these proteins can contribute to cancer progression and metastasis in various types of cancers, such as colorectal carcinoma, different subtypes of breast cancer (triple negative, HER2+, basal, etc.), lung adenocarcinoma, and leukaemia, among others (Belsches-Jablonski et al., 2001; Aligayer et al., 2002; Li, 2007; Zhang et al., 2007; Tryfonopoulos et al., 2011; Chougule et al., 2016; Ichihara et al., 2017). However, oncogenic mutations that directly increase the catalytic activity of SFKs are rare (Irby and Yeatman, 2000; Dehm and Bonham, 2004). Even though there is a correlation between kinase activation and SFK overexpression in tumour biopsies, normal cells do not undergo cell transformation only by SFK overexpression (Gilmer et al., 1985; Anbalagan et al., 2012). Instead, increased activation of SFKs is primarily caused by dysregulation of upstream proteins in the signalling pathway that controls SFK activation and inhibition.

**FIGURE 2:**
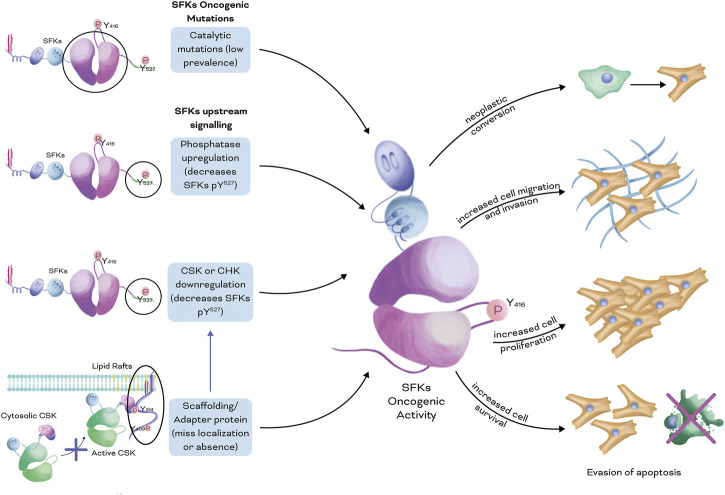
Dysregulated mechanisms that lead to increased activation of SFKs in cancer. Four main mechanisms can lead to increased SFK oncogenic activity: 1. Mutations that increase the catalytic activity of the kinase domain, with low prevalence in patients; 2. Dysregulation of the phosphatases that target the inactivation loop at the C-terminal (Y^527^ for Src or equivalent QYQ peptide); 3. Downregulation of kinases that target the inactivation loop of SFKs, such as CSK and CHK; and 4. Downregulation in the expression levels or mislocalisation of scaffolding or adapter proteins, which allows efficient positioning of CSK or CHK near its SFK substrates. Overall, individually or a combination of these mechanisms leads to increased SFK oncogenic activity, including neoplastic conversion of cells, increased migratory and invasive phenotypes, enhanced cell proliferation, and resistance to apoptotic mechanisms.

Dysregulation of different mechanisms can lead to increased activation of SFKs (Figure 2), including: 1. Dysregulation of phosphatases that target the inactivation loop at the C-terminus (Y^527^ for Src or the equivalent peptide QYQ) (Fang et al., 1994; Roskoski, 2005; Zhu et al., 2007; Narla et al., 2018), 2. Downregulation of proteins like CSK or CHK, which normally phosphorylate the inactivation loop (Chan et al., 2010), 3. Dysregulation of the expression or localisation of scaffolding or adapter proteins that help position SFKs near their substrates (including CSK) (Oneyama et al., 2008; Chüeh et al., 2021), or 4. Dysregulation of kinases/phosphatases that target phosphorylation sites on scaffolding/adapter proteins recognized by the SH2/SH3 domains present in SFKs (Narla et al., 2018). In summary, while SFKs are proto-oncogenes that can contribute to cancer progression, their over-activation is typically not caused by direct mutations in SFKs themselves, but rather by dysregulation of upstream proteins in their signalling pathway.

### 3.1 Crosstalk between integrins and SFKs

**FIGURE 3:**
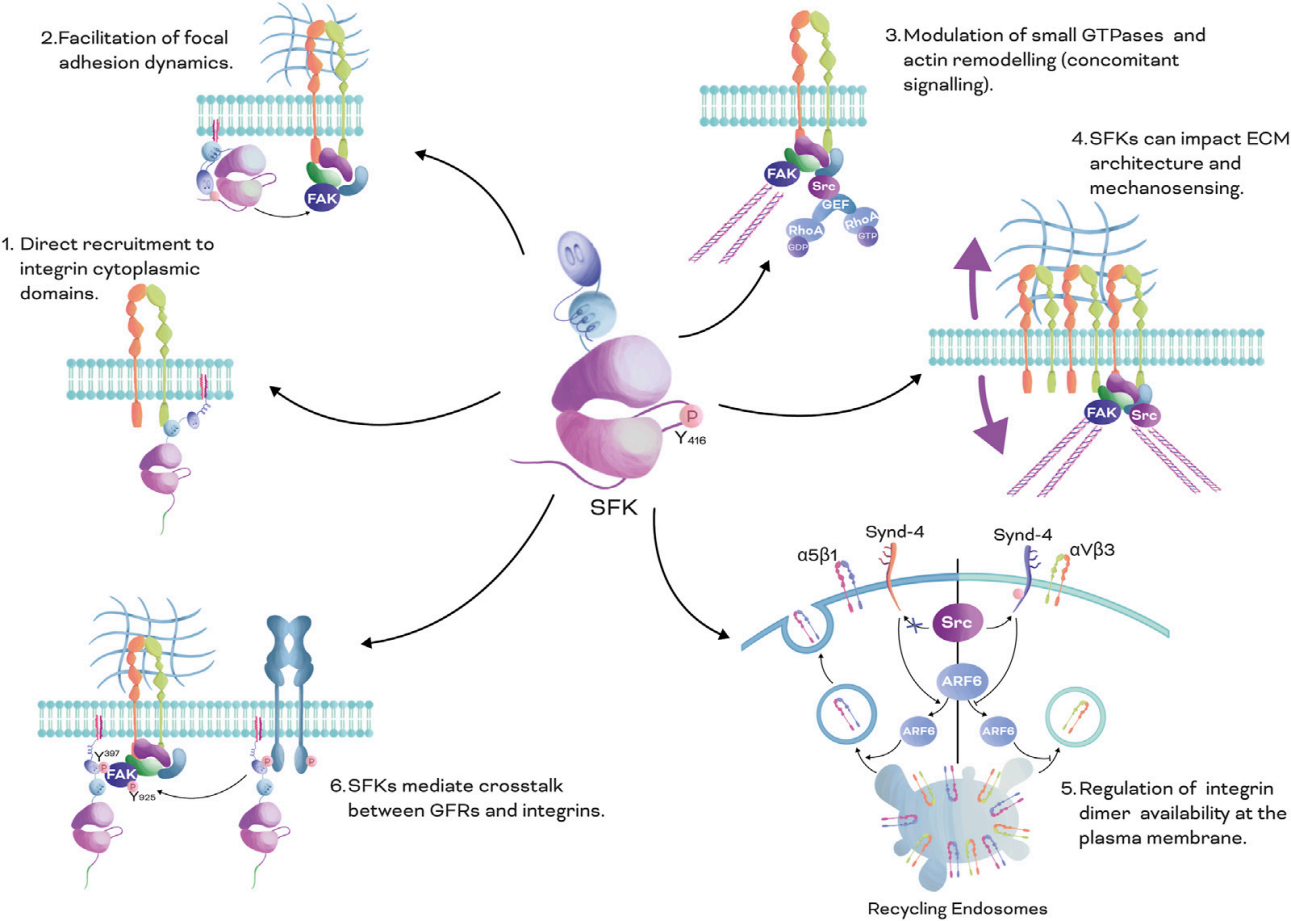
SFKs Mechanisms in Integrin Signalling: There are six main mechanisms by which SFKs mediate integrin signalling. **1.** Direct recruitment to integrin cytoplasmic domains: SFKs can be directly recruited to the cytoplasmic domains of integrins through the SH3 domain, leading to activation of downstream signalling pathways. **2.** Facilitation of focal adhesion dynamics: SFKs can affect focal adhesion dynamics by facilitating the recruitment of adapter and scaffolding proteins, and by forming the Src-FAK phosphorylated complex. This complex can regulate the stability and turnover of focal adhesions, which is important for cell adhesion and migration. **3.** Modulation of small GTPases and actin cytoskeleton dynamics: SFKs can modulate the recruitment and activation of small GTPases, which play crucial roles in regulating actin cytoskeleton dynamics, impacting cell shape, motility, and adhesion. **4.** Impact on ECM architecture and mechanical cues: SFKs can impact the architecture of the ECM, which forms the cellular microenvironment, and respond to mechanical cues, such as matrix stiffness and tension, regulating integrin signalling and cellular behaviour. **5.** Regulation of integrin dimer availability at the plasma membrane: SFKs can regulate the availability of specific integrin dimers at the plasma membrane through recycling and endocytosis processes, influencing integrin-mediated cell adhesion and signalling. **6.** Crosstalk between GFRs and integrins: SFKs can mediate crosstalk between GFRs and integrins, leading to integrated signalling pathways that regulate various cellular processes, such as cell survival, proliferation, and migration. Overall, this figure illustrates the diverse mechanisms by which SFKs play a crucial role in mediating integrin signalling, highlighting their significance in cellular processes and their potential as therapeutic targets for various diseases.

The oncogenic activity of SFKs in cancer has recently been thoroughly investigated in several tissues (Gilmer et al., 1985; Han et al., 1996; Dehm and Bonham, 2004; Playford and Schaller, 2004; Tryfonopoulos et al., 2011). Integrins can crosstalk with SFKs, impacting cellular functions and cancer progression. In short, SFKs can regulate integrin functions through six primary mechanisms (Figure 3). Firstly, SFKs can be directly recruited to the cytoplasmic domains of integrins (Thomas and Brugge, 1997; Zhou et al., 2003; Webb et al., 2004; Sawada et al., 2006; Schwartz and DeSimone, 2008; Watanabe et al., 2009). Secondly, SFKs can affect focal adhesion dynamics by facilitating the recruitment of adapter and scaffolding proteins and by forming the Src-FAK complex (Webb et al., 2004; Mitra and Schlaepfer, 2006; Wan et al., 2017). Thirdly, SFKs can modulate the recruitment/activation of small GTPases and actin cytoskeleton dynamics (Schwartz and Shattil, 2000). Fourthly, SFKs can impact ECM architecture and respond to mechanical cues (Si et al., 2017; Wullkopf et al., 2018; Lamar et al., 2019). Fifthly, they can regulate the availability of specific integrin dimers on the surface of the plasma membrane through recycling and endocytosis (Morgan et al., 2013). Sixthly, SFKs can mediate the crosstalk between GFR and integrins (Li et al., 2016; Ichihara et al., 2017; Thomas et al., 2018; Javadi et al., 2020). In the next sections, we will explore some of these mechanisms.

### 3.2 SFKs and integrin signalling

Integrins regulate cell adhesion and motility via tyrosine kinases. Src can directly interact with the αVβ3 integrin intracellular domain (beta subunit tail) through its SH3 domain (Calderwood et al., 1999; Shattil, 2005) (Figure 3.1). Clustering of αVβ3 integrin activates Src by promoting autophosphorylation of its regulatory loop at Y^416^. Other SFKs, including Hck, Lyn, and Yes, can also be recruited and stabilised by the β1, β2, and β3 integrin cytoplasmic tails.

Integrin engagement and clustering trigger autophosphorylation of FAK at Y^397^, which phosphorylates adapter proteins, such as Paxillin, at the adhesion sites, generating binding sites that SFKs can recognize via their SH2 domains (Webb et al., 2004; Wan et al., 2017; Zhou et al., 2021). For example, Src can be recruited to Paxillin upon FAK phosphorylation; once Src is recruited, it phosphorylates other FAK tyrosine residues, such as Y^925^, increasing FAK activity and forming a FAK-Src complex (Figure 3.2) that phosphorylates adapter proteins, such as p130Cas, resulting in activation of Rac1 and increased cell motility in fibroblasts and epithelial cells (breast cancer) (Harte et al., 1996; Sawada et al., 2006; Zhou et al., 2021). Remarkably, the FAK-Src phosphorylated complex is relevant in tumour growth and metastasis by promoting VEGF-associated angiogenesis and cell invasion (Playford and Schaller, 2004; Mitra and Schlaepfer, 2006).

Particularly relevant is the downstream role of SFKs in the crosstalk between integrins and small GTPases (Clark et al., 1998; Huveneers and Danen, 2009b), allowing integrins to sense and respond to mechanical cues (Figure 3.3). The Rho family of small GTPases is regulated by Guanine nucleotide exchange factors (GEFs), GTPase-activating proteins (GAPs), and Guanosine nucleotide dissociation inhibitors (GDIs). SFKs located downstream of integrins exert control over the activation of these GTPases by influencing the recruitment and activation of GEFs and GAPs (Huveneers and Danen, 2009a). For example, engagement of integrins by Fibronectin via its RGD sequence mediates p190RhoGAP (a negative regulator of RhoA) phosphorylation by Src, promoting cell contraction and traction force (Figure 3.4) (Arthur and Burridge, 2001; Yee et al., 2001). The FAK-Src complex can additionally mediate the recruitment of other GEFs and GAPs that regulate activation of RhoA, Cdc42, and Rac1, including: ArfGAP Paxillin-Kinase Linker (PKL), PAK-interacting exchange factor-beta (β-Pix), Tiam 1, Vav1-3, p190RhoGAP, p250RhoGAP, and p190RhoGEF, among others (Kuroiwa et al., 2011). Integrins can also interact with and control SFKs that are recruited nearby by other membrane scaffolding or adapter proteins. For example, Fyn can be recruited by Caveolin 1 during oligodendrocyte differentiation (Wary et al., 1998; Liang et al., 2004). Once Fyn is recruited, integrin engagement activates Fyn, which decreases RhoA activation by phosphorylating p190RhoGAP (Liang et al., 2004). Thus, Integrins can interact with both, SFKs recruited directly to integrins, and SFKs recruited to other nearby membrane proteins, thus influencing the activation of small GTPases and downstream signalling.

### 3.3 Integrin mechanosensing and SFKs

Integrins respond to external forces by remodelling the actin cytoskeleton through activation of SFKs and small GTPases. For example, in response to increased Fibronectin rigidity, αVβ3 integrin increases Src and RhoA activity in cancer cells (Knowles et al., 2013; Daday et al., 2022). In diverse cancer types, matrix stiffness is increased in tumours and can be sensed by integrins, promoting more invasive phenotypes (Gkretsi and Stylianopoulos, 2018; Wullkopf et al., 2018; Deng et al., 2022). In addition, reorganisation of the cytoskeleton directly impacts pro-oncogenic pathways involved in cell proliferation and survival, such as PI3K/AKT, Ras/ERK, Smads, Yes-associated protein (YAP) and the transcriptional co-activator with PDZ-binding motif (TAZ), among others, leading to cell reprogramming and neoplastic conversion (Hanahan and Weinberg, 2000; Nardone et al., 2017; Block et al., 2020). Increased ECM stiffness promotes RhoA activation and cell contractility via Src kinase recruitment, initiating differentiation of breast cancer epithelial cells (Reid et al., 2017). Furthermore, sustained ECM rigidity triggers changes in the expression, repertoire, conformation (activation) and clustering of integrins, generating a positive loop of activation (Figure 3.4) that maintains exceeded cell proliferation of tumour cells.

Integrins sense differences in ECM rigidity and orchestrate deposition, fibrillogenesis, and degradation of the ECM (Balcioglu et al., 2015; Hamidi and Ivaska, 2018). Crosstalk between integrins, small GTPases and SFKs is required to finely tune ECM assembly; for example, engagement of α5β1 integrin with soluble Fibronectin promotes Fibronectin fibrillogenesis by increasing Src/RhoA-mediated cytoskeleton contraction (Danen et al., 2002). Cell contraction and α5β1 integrin translocation from focal adhesions to fibrillar adhesions causes stretching of soluble Fibronectin dimers, initiating matrix assembly. Furthermore, Fibronectin assembly in the ECM is required prior to the initiation of Collagen fibrillogenesis and organisation by Collagen-binding integrins, suggesting that Collagen matrix assembly is also controlled by the crosstalk between integrins and SFKs. Remarkably, changes in ECM stiffness can drive neoplastic conversion of cells, changing the expression levels and repertoire of integrin heterodimers (Hamidi and Ivaska, 2018). Thus, SFK regulation of ECM fibrillogenesis also impacts integrin expression and availability.

One central pathway, which enables the cell to respond to mechanical cues, is the Hippo pathway (Nardone et al., 2017; Totaro et al., 2018). The adhesome machinery transduce those mechanical cues from the extra-to-intracellular compartments, by promoting the translocation of YAP/TAZ from the cytoplasm to the nuclei, thereby modulating gene expression (Nardone et al., 2017; Totaro et al., 2018; Rausch and Hansen, 2020). YAP/TAZ nuclear translocation and activity are regulated by ECM composition/rigidity, cell density and cell shape (Totaro et al., 2018; Scott et al., 2021). Particularly, crosstalk between β1 integrins and SFKs (e.g., Src, Fyn, and Yes) is required for YAP nuclear translocation (Totaro et al., 2018; Lamar et al., 2019; Block et al., 2020). β1 integrins via SFKs can promote Rac1 activation at cell protrusions, leading PAK1 downstream signalling to phosphorylate Merlin. Merlin, YAP, and LATS form an inhibitory complex that is localised at the cytoplasm; when Merlin is phosphorylated, YAP is released from this complex, allowing its translocation to the nucleus. Once at the nucleus, YAP/TAZ interact with TED family members, regulating gene transcription (Si et al., 2017). Remarkably, Src-mediated YAP/TAZ activation is an important driver of cancer progression in breast and melanoma cells (Lamar et al., 2019; Ding et al., 2021; Ortega et al., 2021). Indeed, Src upregulation (by activating ArfGAP1 and repressing LATS) significantly increases YAP/TAZ activity, as well as modulates the expression of YAP/TAZ-regulated genes, such as CTGF, TGFB1, EGFR, SOX2 (SRY-Box Transcription Factor 2), BIRC5 (Survivin), CTNNB1 (β-Catenin), and FGF1/2 (Fibroblast Growth Factor 1 and 2), among others, promoting tumour growth and metastasis (Zhao et al., 2008; Muramatsu et al., 2010; Frum et al., 2018; Lu et al., 2018; Qin et al., 2018; Ortega et al., 2021; Lu et al., 2022).

Integrin mechanotransduction can mediate SFK recruitment to adhesion sites. For example, β1 integrins can sense laminar shear stress, triggering Caveolin-1 phosphorylation at Y^14^ and recruitment of CSK to adhesion sites in aortic endothelial cells (Radel and Rizzo, 2005). Indeed, β1 integrin blockade with antibodies inhibits SFK and p190RhoGAP phosphorylation observed upon shear stress. Furthermore, Caveolin-1 depletion avoids p190RhoGAP phosphorylation caused by sustained SFK activity, and both Caveolin-1 or β1 integrin inhibition disrupts shear regulation of RhoA (Radel and Rizzo, 2005). Caveolin-1-mediated recruitment of CSK at the β1 integrin sites is necessary for inducing Myosin Light Chain (MLC) dephosphorylation, which explains how hemodynamic shear stress influences the endothelial cell phenotype. In addition, MLC phosphorylation via RhoA activation is a well-known mechanism that generates contractile force in cells such as fibroblasts (Yee et al., 2001). Thus, CSK recruitment may also reduce the traction force generated by aortic endothelial cells in response to laminar shear stress; however, this mechanism has not been studied yet.

In summary, integrins play a crucial role in sensing and responding to changes in ECM rigidity, which can impact cell behaviour and promote tumour progression (Gkretsi and Stylianopoulos, 2018). Integrins can activate SFKs and small GTPases, leading to cytoskeleton remodelling, changes in gene expression, and alterations in ECM assembly. The crosstalk between integrins and SFKs is also involved in regulating YAP/TAZ nuclear translocation and activity, which can drive cancer progression (Reid et al., 2017; Totaro et al., 2018; Lamar et al., 2019). Furthermore, integrins can mediate SFK recruitment at adhesion sites, affecting downstream signalling pathways, such as RhoA and MLC. Overall, the integrin-SFK axis is a promising target for therapeutic interventions in cancer and other diseases associated with alterations in ECM stiffness.

### 3.4 SFKs and integrin trafficking

Receptor availability on the surface of the plasma membrane is an important mechanism of cell signalling regulation in cancer cells. Integrin trafficking, including internalisation, recycling, and degradation, controls the amount of receptor available at the cell surface to sense and respond to extracellular cues (Chao and Kunz, 2009; Bridgewater et al., 2012; Paul et al., 2015). Spatiotemporal coordination of adhesion complex dynamics is key for important cellular processes, including migration, invasion, cell differentiation and response to growth factors, among others. In this section, we will summarize how SFKs contribute to control integrin availability at the plasma membrane through an endocytic mechanism (Figure 3.5).

Clathrin-mediated endocytosis is a well-characterised pathway for internalisation of cell surface receptors, including integrins (Ezratty et al., 2009; Paul et al., 2015). It involves the formation of Clathrin-coated pits at the plasma membrane, which bud off to form Clathrin-coated vesicles containing the internalised receptors. SFKs can play a role in initiating this process by phosphorylating tyrosine residues on the cytoplasmic domain of integrins, which can directly or indirectly recruit adaptor proteins, such as AP2, Dab2, and Eps8, to the plasma membrane, and facilitate the formation of Clathrin-coated pits (Chao and Kunz, 2009; Ezratty et al., 2009; Paul et al., 2018). Furthermore, SFKs can also modulate the recruitment/activation of other signalling proteins involved in Clathrin-dependent endocytosis of integrins, including ARFGTPases (e.g., ARF6) (Heckel et al., 2009; Morgan et al., 2013).

Adherent cells that bind Fibronectin differentially engage α5β1 and αVβ3 integrins to allow changes in mechanosensing, adhesion complex stability, and matrix assembly. Thus, controlling the membrane surface levels of these proteins impacts cell migration and ECM architecture (Paul et al., 2015). Syndecan-4 is a membrane-intercalated heparan sulfate proteoglycan receptor that can interact with the ECM (Fibronectin) and cell adhesion molecules (for example, Thy-1), and that also acts as a co-receptor for GFRs, integrins, cytokines, and morphogens (Elfenbein and Simons, 2013). Syndecan-4 can be phosphorylated at Y^180^ by Src, controlling cell adhesion dynamics, cell migration and integrin recycling (Morgan et al., 2013) (for example, of α5β1 and αVβ3 integrins). Syndecan-4 allows the recruitment of Syntenin, suppresses Arf6 activity, and enhances the recycling of αVβ3, limiting α5β1 integrin recycling to the plasma membrane (Figure 3.5) (Morgan et al., 2013). In addition, increased levels of αVβ3 integrin at the plasma membrane promote stabilisation of focal adhesion complexes. On the contrary, abrogation of Src-dependent phosphorylation of Syndecan-4 results in increased surface expression of α5β1 integrin and destabilisation of focal adhesion complexes.

In summary, SFKs play a crucial role in regulating integrin availability in the plasma membrane through endocytic mechanisms, such as Clathrin-mediated endocytosis and Syndecan-4 mediated recycling (Bridgewater et al., 2012; Morgan et al., 2013). These mechanisms are essential for controlling cellular processes, such as migration, invasion, differentiation. Therefore, the spatiotemporal coordination of focal adhesion complex dynamics is a critical mechanism for maintaining cellular homeostasis and proper cellular functions.

## 4 Regulation OF CSK and integrins in cancer

Sustained activation of SFKs exerts a well-known oncogenic activity by impairing mechanisms that control integrin and SFK crosstalk (Figure 3), impacting normal cellular functions. Although SFK mutations that can increase the catalytic activity of these kinases (Irby et al., 1999; Turro et al., 2016), they are rare in patients, and most of the reported evidence suggests that increased activation of SFKs is primarily caused by dysregulation of upstream signalling pathways, such as CSK-dependent inhibition of SFKs (Figure 2) (Bénistant et al., 2001; Pema et al., 2005; Chougule et al., 2016; Chüeh et al., 2021).

Accumulating evidence supports the key role that CSK may play in inhibiting cancer progression by downregulating SFK oncogenic activity (Masaki et al., 1999; Nakagawa et al., 2000; Bénistant et al., 2001; Kuga et al., 2020). Indeed, activation of Src via CSK downregulation increases cell proliferation and angiogenesis in pancreatic cancer (Masaki et al., 1999). In pancreatic cancer cells, CSK inhibition using the inhibitor ASN2324598 substantially decreases Y^527^ phosphorylation in Src, resulting in increased proliferation and activation of pro-oncogenic pathways, such as MAPK/MEK, and pro-angiogenic growth factors, like VEGF (Pema et al., 2005). Furthermore, overexpression of CSK in human colon cancer cells (adenocarcinoma) inhibits tumour growth (Nakagawa et al., 2000). In addition, CSK overexpression abrogates the highly metastatic phenotype of NL-17 cells *in vitro*, resulting in suppression of metastasis in mouse xenografts (Nakagawa et al., 2000).

Overall, integrins can crosstalk with SFKs, influencing cellular functions and cancer progression. Given this context, dysregulation of CSK, one of the most crucial negative regulators of SFKs, may impact the underlying mechanisms that drive sustained SFK activation and integrin signalling. In this section, we will delve into the structure and regulation of CSK and examine some specific examples that demonstrate how CSK may regulate cancer progression through integrin/SFK signalling.

### 4.1 CSK structure and regulation

CSK is a well-known member of SFKs that suppresses SFK activity by phosphorylating Y^527^ or an equivalent in its C-terminal negative regulatory loop (Okada et al., 1991; Ia et al., 2010). CSK is ubiquitously expressed in all cell types and possesses a similar structural arrangement to that of other SFKs, including SH3 and SH2 domains in its N-terminus and a kinase domain in its C-terminus (Figure 1A). However, CSK lacks both the classical autophosphorylation site in the activation loop and the N-terminal fatty acylation sites (Figure 1A) that allow other family members, such as Src, Fyn, and Lyn, among others, to be anchored to the plasma membrane (Okada et al., 1991; Sabe et al., 1994; Cole et al., 2003; Ia et al., 2010). Thus, CSK is mainly located in the cytosol and its regulation differs from other SFKs; CSK recruitment to the plasma membrane is regulated by scaffolding or adapter proteins that are located in close proximity to other SFKs (Howell and Cooper, 1994; Cole et al., 2003; Lee et al., 2006).

CSK binds scaffolding and adapter proteins via its SH2 domain, which recognizes phosphotyrosine-containing peptide sequences that recruit CSK to the plasma membrane (Sabe et al., 1994; Cole et al., 2003; Ia et al., 2010). Remarkably, even though CSK is constitutively active (basal levels), disruptions in the SH2 domain greatly impact CSK catalytic activity. Deleting the SH2 domain or crosslinking cysteines in the SH2 domain inhibits CSK activity, revealing that the SH2 domain is not only relevant for recruiting CSK near SFKs but also for fully activating its kinase domain (Cloutier et al., 1995; Dias et al., 2022). In fact, engagement of the SH2 domain with the phosphorylated sites of adapter proteins highly increases CSK catalytic activity (Sondhi et al., 1998). In addition, the oxidation state of the disulfide bond present in the SH2 domain can also impact the kinase activity of CSK (Sondhi et al., 1998; Mills et al., 2007; Ia et al., 2010; Liu and Cowburn, 2016). Analysis of both oxidized and reduced forms of the CSK-SH2 domain by NMR revealed two distinctive conformational states of the kinase, suggesting that cell redox pathways may also regulate CSK activity (Mills et al., 2007). CSK can also be phosphorylated by Protein Kinase A (PKA) at Serine 364, increasing its kinase activity by 2-4-fold (Abrahamsen et al., 2003; Yaqub et al., 2003). The interaction between PKA and the SH3 domain of CSK, as well as an overall re-arrangement of the structure of the kinase domain, appears to be responsible for this enhanced activation (Yaqub et al., 2003).

CSK can be recruited to the plasma membrane in a variety of cellular contexts by several scaffolding/adapter proteins and receptors, including: Caveolin-1, Lck-interacting Membrane Protein (LIME), Signalling Threshold Regulating Transmembrane Adaptor 1 (SIT1), Clathrin Adaptor Protein (Dab2), VE-Cadherin, Insulin Like Growth Factor 1 Receptor (IGF-1R), Insulin receptor, Paxillin, and CBP, among others (Sabe et al., 1994; Tobe et al., 1996; Arbet-Engels et al., 1999; Kawabuchi et al., 2000; Pfrepper et al., 2001; Cao et al., 2002; Brdicková et al., 2003; Zhou et al., 2003; Baumeister et al., 2005; Maldonado et al., 2017). Remarkably, some of these transmembrane proteins, such as CBP, possess multiple phosphorylation sites that can be recognized by SH2 domains-containing proteins, allowing the scaffold protein to simultaneously bind to more than one SFK and enabling efficient inactivation of SFKs by recruiting CSK within the same scaffold complex (Brdicka et al., 2000; Kawabuchi et al., 2000).

CSK substrate specificity is not only related to the recognition of a QYQ peptide sequence in SFKs, but also depends on a three-dimensional arrangement between SFKs and CSK, not involving either the SH2 or SH3 domains (Cloutier et al., 1995; Sondhi et al., 1998; Young et al., 2001; Lee et al., 2006). In *in vitro* experiments with Src and CSK, it has been shown that the QYQ sequence is crucial for phosphorylation but not sufficient to explain the interactions of these proteins; in fact, CSK catalytic activity is relatively low with mutants that only recognize the QYQ peptide. In addition, the Y^511^ residue in the Src tail might be relevant in this process, since mutations result in a 50% drop of Src phosphorylation by CSK (Lee et al., 2006). Furthermore, other proteins, such as Paxillin, LATS, c-Jun and the P2X3 receptor, can serve as CSK substrates (Zhu et al., 2006). However, the role that CSK plays in the regulation of these proteins remains elusive.

CSK is a highly selective tyrosine kinase compared to other family members, such as Src, which shows a broader recognition ability for tyrosine-containing peptide sequences (Lee et al., 2006; Levinson et al., 2008; Maldonado et al., 2017). Once CSK is recruited near potential substrates by scaffolding or adapter proteins, it recognizes a specific QYQ sequence at the C-terminus and phosphorylates a single tyrosine residue in SFK members, causing conformational changes and inactivating them. In the next section, we will revise how CSK interacts with SFKs and expand how CSK-dependent inhibition of SFKs can impact integrin signalling in cancer.

### 4.2 CSK and cell adhesion signalling

CSK can define integrin-SFK-mediated cell adhesion signalling, which can also impact the metastatic potential of cancer cells. For example, CSK defines integrin-mediated cell adhesion and migration in human colon cancer cells (Rengifo-Cam et al., 2004; Webb et al., 2004). CSK dominant-negative constructs expressed in cells lead to increased SFK activation, which promotes the phosphorylation of FAK and Paxillin, two key components of focal adhesion complexes. Consequently, this results in an increase in the size/number of focal adhesions, and promotes more cell adhesion, actin cytoskeleton rearrangement, and enhanced cell migration and invasiveness. Furthermore, CSK can localise in focal adhesions via adapter/scaffolding proteins. For example, overexpressed CSK is localised in focal adhesions via Paxillin, causing αVβ5 reorganisation and changes in spreading of HeLa cells (Bergman et al., 1995).

Other downstream effectors of integrins can also be regulated by CSK. For example, in fibroblasts, Integrin-mediated activation of ERK is downregulated upon adhesion to Fibronectin or Laminin in cells with silenced CSK, whereas AKT activation is increased (Gu et al., 2003). Therefore, CSK (−/−) cells are more resistant to serum-induced apoptosis but are less proliferative in compassion to WT cells (Gu et al., 2003). These results suggest that CSK can differentially modulate integrin downstream signalling. Furthermore, overexpression of CSK suppresses stretch-induced activation of Src and p38 MAPK, whereas overexpression of a kinase-negative CSK construct has no effect in cardiomyocytes (Aikawa et al., 2002).

Other tyrosine kinases (outside of SFK family) can also be regulated by CSK/SFKs influencing integrin signalling, one example of this is the Spleen Tyrosine Kinase (SYK) (Obergfell et al., 2002). SYK is expressed mainly in hematopoietic cells (leukocytes, platelets) but also in other tissues including liver (hepatocytes, hepatic stellate cells, Kupffer cells) and heart (Sada et al., 2001; Qu et al., 2018; Li et al., 2023). In platelets, fibronectin binding to αIIbβ3 integrin results in CSK exclusion from the integrin adhesion sites, increasing Src activation (Obergfell et al., 2002). Remarkably, Src inhibition precludes SYK activation and the phosphorylation of SYK substrates (Vav1, Vav3, SLP-76) involved in actin cytoskeleton regulation (Obergfell et al., 2002). Indeed, SYK-deficient platelets exhibited Src activation upon adhesion to fibrinogen, but no spreading or phosphorylation of Vav1, Vav3, and SLP-76 (Obergfell et al., 2002). Furthermore, SYK is also activated during integrin signalling transduction in leukocyte, resulting in increased leukocyte adhesion on endothelial cells (Vines et al., 2001; Mócsai et al., 2006; Frommhold et al., 2007). Also, CSK-deficient granulocytes exhibit increased degranulation and integrin signalling activation (Thomas et al., 2004). Consequently, granulocytes show hyperphosphorylation of integrin downstream effectors including SYK and Paxillin, showing a more adhesive phenotype with impaired migration (Thomas et al., 2004).

In summary, CSK plays a crucial role in defining integrin-SFK-mediated cell adhesion signalling, which may significantly impact the metastatic potential of cancer cells. CSK can regulate integrin downstream effectors, such as FAK, Paxillin, ERK, AKT, and SYK, resulting in different cellular responses. CSK can also localise directly in focal adhesions via adapter proteins, causing reorganisation of integrins and impacting cancer progression.

### 4.3 CSK subcellular localisation

Spatiotemporal localisation of proteins is one of the main mechanisms though which cells can control biological processes. CSK does not possess an N-terminal fatty acylation site, which allows other family members to be anchored to the plasma membrane, and therefore, requires recruitment near SFKs by adapter or scaffolding proteins (Figure 1A) (Okada et al., 1991; Levinson et al., 2008). Consequently, mislocalisation of CSK has been studied as a potential mechanism to dysregulate the oncogenic activity of SFKs. For example, SFK-driven colon cancer cell invasion is induced by dysregulation of CSK membrane localisation (Kawabuchi et al., 2000; Oneyama et al., 2008). In colon cancer cells, CSK expression levels were inconsistent with SFK activity; nevertheless, CSK translocation to the plasma membrane was impaired by downregulation of its scaffolding protein CBP (Sirvent et al., 2010). Thus, re-expression of CBP inhibited SFK oncogenic activity and cell invasion in a CSK-dependent manner.

CBP expression is downregulated in several types of cancer cells and may interfere with CSK translocation to the plasma membrane (Oneyama et al., 2008; Agarwal et al., 2016). Indeed, low expression of CBP is a prognostic factor indicating worse survival, increased relapse, and advanced stage disease in neuroblastoma patients (Agarwal et al., 2016). CBP is also downregulated in non-small lung cancer cells, which show upregulation of c-Src (Kanou et al., 2011). Moreover, ectopic CBP expression suppresses anchorage-independent growth of A549 and Lu99 cell lines (non-small lung cancer) and suppresses c-Src activity by recruiting CSK to lipid rafts in A549 cells (Kanou et al., 2011). In addition, CBP expression reduces *in vitro* invasion and the ability of A549 cells to form tumours in nude mice (Kanou et al., 2011).

In neoplastic converted cells, CBP downregulated expression may be modulated by epigenetic histone modifications via MAPK/PI3K pathways (Suzuki et al., 2011). Oncogenic Src- and Ras-transformed fibroblasts show reduced levels of CBP and enhanced MEK and AKT activity. Remarkably, Src-mediated transformation did not affect the stability of CBP mRNA, transcriptional activity of the CBP promoter, nor DNA methylation of the CBP promoter CpG islands (Suzuki et al., 2011). On the contrary, Src-mediated transformation decreased histone H4 total acetylation levels (analysed by western blot) and increased histone H3 methylation (K^27^) levels in the CBP promoter (Suzuki et al., 2011). Consequently, inhibition of MEK, PI3K, or histone deacetylases restored CBP expression levels, suggesting that CBP downregulation may be mediated by epigenetic histone modifications via oncogenic MAPK/PI3K pathways (Suzuki et al., 2011).

As we previously mentioned, Caveolin-1 can also recruit CSK to the plasma membrane, playing a relevant role by controlling the SFK/p190RhoGAP/RhoA axis and the response to mechanical stress (hemodynamic shear stress) (Radel and Rizzo, 2005). Interestingly, in lung fibroblasts of Caveolin-1 knockdown mice, Src activity and CSK membrane localisation were similar to the WT mice (Place et al., 2011). On the contrary, CBP expression was increased in comparison to the WT mice. Remarkably, CBP deletion in WT cells did not influence Src activity but increased Caveolin-1 phosphorylation at Y^14^, which is required for CSK recruitment. Knockdown of CBP by siRNA in Caveolin-1 KO cells increased Src activity, and re-expression of WT Caveolin-1 in the same cells reduced Src activity. These results suggest that Caveolin-1 and CBP cooperatively regulate Src activity by recruiting CSK to the membrane and this may be a compensatory mechanism when either Caveolin-1 or CBP expression is reduced (Place et al., 2011). However, there are still many questions about this model that need to be explored in the future, considering that in other models of cancer, such as neuroblastoma, CBP downregulation appears to be enough to drive Src activation. A different possibility is that the role of Caveolin-1 is more prominent regulating SFKs via CSK by mechanical stress cues, but no such studies are available yet.

In conclusion, CSK, a critical negative regulator of SFK activity, requires recruitment to the plasma membrane to effectively regulate SFKs. Dysregulation of CSK localisation can contribute to oncogenic activity of SFKs, leading to cancer cell invasion and tumour formation (Figure 2). CBP, a scaffolding protein involved in recruiting CSK to the membrane, is downregulated in several types of cancer cells, contributing to SFK activation. Mechanisms that modulate CBP expression, including epigenetic histone modifications via the MAPK/PI3K pathways, have been identified (Kawabuchi et al., 2000; Xu et al., 2005; Oneyama et al., 2008; Suzuki et al., 2011). Additionally, Caveolin-1 can recruit CSK to the plasma membrane, and a cooperative relationship between Caveolin-1 and CBP in regulating SFK activity has been proposed. Understanding the complex interplay between these regulatory mechanisms may provide insights into potential therapeutic targets for cancer treatment.

### 4.4 CSK and growth factor receptors

The transmembrane GFRs engage growth factors (e.g., EGF, PDGF, and TGFβ) and transduce signals intracellularly (McInnes and Sykes, 1997; Wieduwilt and Moasser, 2008; Tiash and Chowdhury, 2015). GFRs control relevant cellular processes, such as cell migration, proliferation, differentiation, and survival. The intracellular domain of GFRs possesses the kinase catalytic activity. Several GFRs, including EGFR, VEGFR, FGFR, PDGFR, IGFR, HER2, and the Insulin Receptor (IR), are Receptor Tyrosine Kinases (RTKs), and others, such as the TGFβ receptor, can transduce signalling via serine-threonine kinases and G-protein-coupled receptors.

GFRs can interact with integrins via 4 general mechanisms: 1. They can interact directly. For example, integrins can transactivate GFRs independently of growth factor ligand engagement by co-clustering of the receptors, promoting GFR autophosphorylation (Moro et al., 2002a; Mitra et al., 2011; Latko et al., 2019; Nozaki et al., 2019). 2. By co-regulating common downstream signalling. Integrins and GFRs share common downstream signalling, such as MAPK, FAK, Src, Ras, SFKs, PI3K, small GTPases (Rho family), Abl, Integrin-linked kinase, ROCK, Smads and YAP/TAZ, among others (Woodard et al., 1998; Somanath et al., 2009). 3. Integrins can activate growth factors (Ivaska and Heino, 2011). For example, αV integrins can activate TGFβ (Ivaska and Heino, 2011). 4. By co-regulating receptor trafficking. For example, EGF causes co-internalization of EGFR/α2β1 in platelets, EGFR/αVβ3 in fibroblasts, and EGFR/αVβ6 in breast cancer cells (Ivaska and Heino, 2011; Thomas et al., 2018). This mechanism allows both families of receptors, integrins and GFRs, to reciprocally co-regulate their surface expression.

SFKs can interact with both GFRs and integrins (Ivaska and Heino, 2011; Maldonado and Hagood, 2021). GFRs, as well as integrins, possess docking sites for SH2 and SH3 domains, respectively, allowing them to interact with adapter proteins and SFKs. GFRs and integrins co-regulate several common pathways, sometimes cooperatively, by amplifying, transactivating, or inhibiting downstream common signalling. For example, Src kinase activity is required for integrin-mediated transactivation of the EGFR (Moro et al., 2002b; Li et al., 2016). On the other hand, Src recruited by activated EGFR can phosphorylate FAK on Y^925^ to further activate its kinase activity downstream of integrins (Javadi et al., 2020) (Figure 3.6).

CSK can also regulate GFRs signalling by inactivating recruited SFKs activated by GFRs (Li et al., 2016). CSK-mediated phosphorylation of SFKs recruited by GFRs leads to the inhibition of downstream signalling pathways co-regulated by integrins, including the Ras/MAPK and PI3K/AKT pathways, resulting in decreased cellular proliferation, migration, and survival (Howell and Cooper, 1994; Nakagawa et al., 2000; Gu et al., 2003; Veracini et al., 2008; Li et al., 2016). For example, CSK-deficient mouse embryonic fibroblast (MEF) cells preclude cell migration induced by PDGF, EGF, and serum, and CSK re-expression rescues the MEF migratory phenotype (McGarrigle et al., 2006). Despite this, CSK deletion did not affect Rac1 activation nor lamellipodia formation, but impaired focal adhesion formation, altering the migratory phenotype in MEFs.

CSK enhances dephosphorylation of focal adhesion proteins induced by GFRs and cytokines. For example, overexpression of CSK enhances and prolongs insulin-stimulated dephosphorylation of pp125FAK and Paxillin (Tobe et al., 1996). Indeed, CSK dead kinase transfection inhibits both p-FAK and p-Paxillin dephosphorylation (Tobe et al., 1996). Furthermore, the ability of CSK to control focal adhesion protein phosphorylation may have a key impact in cancer progression and metastasis. Indeed, many cancer cells express low levels of CSK and elevated activation of SFKs, leading to more invasive cell phenotypes (Howell and Cooper, 1994; Han et al., 1996; Obergfell et al., 2002; Pema et al., 2005; Wheeler et al., 2009; Li et al., 2016; Wullkopf et al., 2018; Ding et al., 2021). The relevance of CBP enabling efficient inactivation of SFKs by CSK has also been proven to be crucial in preventing tumorigenesis and controlling GFR signalling in cancer (Jiang et al., 2006; Gargalionis et al., 2014; Li et al., 2016; Nozaki et al., 2019).

Another key target of CSK is the GFR-bound protein 2 (Grb2)-associated binder 1 (Gab1) protein, which is a critical mediator of signalling pathways downstream of RTKs (Kiyatkin et al., 2006; Hoeben et al., 2013). Gab1 is an adapter protein that interacts with various signalling molecules, including PI3K, SFKs, and the MAPK pathway (Yart et al., 2001; Schaeper et al., 2007). These interactions may lead to the activation of downstream signalling cascades that regulate cell growth, proliferation, migration, and survival (Schaeper et al., 2007). CSK has been shown to regulate Gab1 by directly phosphorylating this protein (Watanabe et al., 2009). This phosphorylation event inhibits the interaction of Gab1 with various signalling molecules, including PI3K and the Ras/MAPK (Watanabe et al., 2009). As a result, the activation of downstream signalling pathways is decreased, which can have profound effects on cell growth and survival (Watanabe et al., 2009).

In conclusion, the regulation of cooperative signalling between GFRs and integrins by SFKs and CSK plays a crucial role in various biological processes, such as cell differentiation, migration, proliferation, and apoptosis. CSK acts as a key regulator by inactivating SFKs recruited or activated by GFRs, inhibiting downstream signalling pathways, and dephosphorylating focal adhesion proteins induced by integrins, GFRs and cytokines. Moreover, CSK also targets the adapter protein Gab1 to modulate signalling cascades involved in cell growth and survival.

## 5 Concluding remarks

Integrins play a central role in focal adhesion complex formation, enabling cells to sense, respond to, and modify their surrounding environment (Humphries et al., 2006; Schwartz, 2010). Precise regulation of the composition and function of focal adhesion complexes is essential for maintaining healthy tissues and normal organ function (Liang et al., 2004; Evans et al., 2009; Keely, 2011; Shen et al., 2021). In cancer cells, the oncogenic activity of SFKs contributes to dysregulation of integrin signalling at various levels, including integrin availability at the cell membrane, activation of growth factors, focal adhesion composition and dynamics, and mechanical response to external cues (Verbeek et al., 1996; Irby et al., 1999; Irby and Yeatman, 2000; Dehm and Bonham, 2004; Kuga et al., 2020). This dysregulation leads to increased invasiveness, migration, uncontrolled cell proliferation, and evasion of normal cell death programs like apoptosis, thus promoting cancer cell survival.

Currently, there are several therapies under development that target integrins and their downstream effectors (Hamidi et al., 2016; Bergonzini et al., 2022). These include the use of antibodies that block specific integrin dimers, such as αVβ1, α5β1, αVβ3, αVβ5, αVβ6, and αVβ8 (Abituzumab, Intetumumab, and Etaracizumab); small molecules (mimetics) and peptides that target the RGD-binding peptide sequence found in alphaV integrins (e.g., Cilengeotide) (Landen et al., 2008; Ning et al., 2010; Wang et al., 2013; Jiang et al., 2017; Ludwig et al., 2021). Other potential drugs include kinase inhibitors that target ERK/AKT, Src (Dasatinib), and RTKs such as GFRs; as well as small molecule inhibitors that target downstream effectors like FAK (defactinib, GSK2256098, VS-6063, and BI 853520), among others (Zhang et al., 2014; Jones et al., 2015; Tiede et al., 2018; Cao et al., 2019; Maiti et al., 2020; Mongre et al., 2021; Wang-Gillam et al., 2022). However, the complex regulation of integrins and their crosstalk with other signalling pathways such as the GFR cascade, combined with the multifactorial nature of neoplastic conversion in different tissues, has hindered the translation of these pharmacological approaches into effective clinical treatments.

**FIGURE 4:**
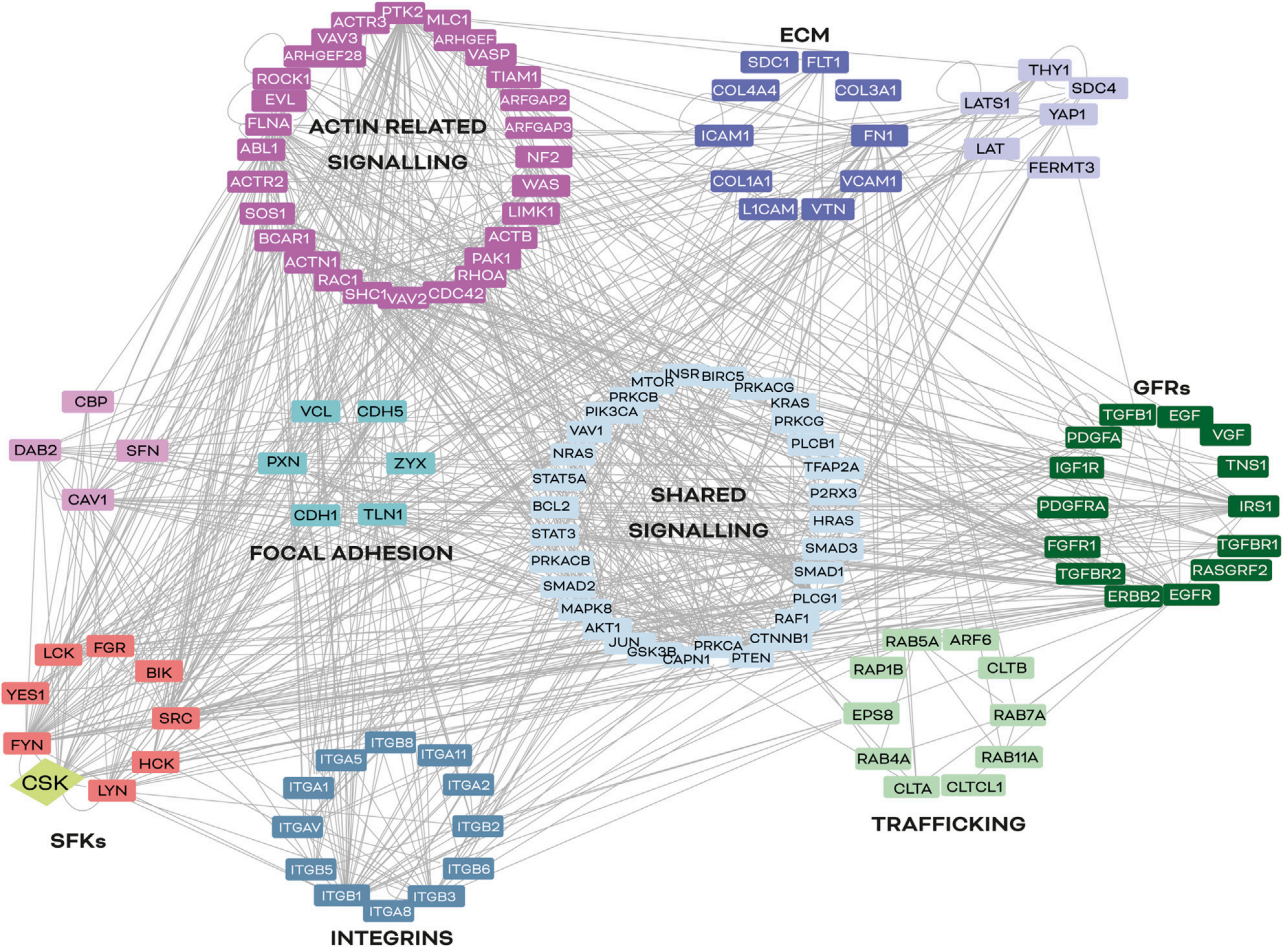
Common signalling nodes between integrins and SFKs in cancer. *In silico* analysis of signalling nodes between integrins and SFKs in cancer. Proteins selected for this map have been described to be involved in cancer progression and integrin signalling regulation in at least two different organs (lung, kidney, skin, heart, and/or liver). Lines represent a described interaction (physical or functional) between the molecules reported in the literature, according to the UniProt database (https://www.uniprot.org). The software used was Cytoscape 3.7.

Although promising therapeutic approaches targeting integrins and their downstream effectors are under development, CSK has emerged as a potential mediator between SFKs and integrin signalling, regulating SFK activity and GFR crosstalk (Okada et al., 1991; Bénistant et al., 2001; Gu et al., 2003; Kunte et al., 2004; Li et al., 2016; Xiao et al., 2018). As shown in Figure 4, CSK is a node of convergence of the intricated signalling pathways that control neoplastic cell conversion and lead to more invasive cell phenotypes. While CSK has shown promising results in reducing tumour growth and metastatic potential in cancer models, further research is needed to fully understand its potential as a cancer therapeutic target. Additionally, the role of adapter and scaffolding proteins that guide CSK to the correct cellular location and timing also requires further investigation. Therefore, identifying convergence points between dysregulated pathways in cancer cells is crucial for developing effective strategies in cancer therapy.
